# Myths, misconceptions, and hesitancy in people residing in Qatar toward mRNA COVID-19 vaccines: An experience exchange from Qatar University health center

**DOI:** 10.5339/qmj.2022.fqac.20

**Published:** 2022-03-30

**Authors:** Maryam Al-Rashid, Areej Al-Hamad, Ahmad Al-Hamad, Yasin Yasin

**Affiliations:** ^1^Primary Health Care Corporation, Academic Affairs Department, Doha-Qatar. E-mail: malrashid@phcc.gov.qa; ^2^Sidra Medicine, Diagnostic Imaging, Doha-Qatar.; ^3^University of Calgary, Doha-Qatar.

**Keywords:** hesitancy, mRNA COVID-19 vaccines, Qatar

## Abstract

The hesitancy in taking COVID-19 vaccines is a complex process influenced by several factors, including individual, social, and cultural. Health literacy and community awareness around mRNA COVID-19 vaccines are critical for successfully combating the pandemic. Healthcare professionals, including family physicians and nurses, can help increase community awareness and mitigate some misconceptions and hesitancy regarding mRNA COVID-19 vaccines in people's attitudes. Therefore, in this study, we aimed to explore how the interaction between an individual's social identities such as gender, ethnicity, culture, knowledge, and belief impact their hesitancy and attitudes toward mRNA COVID-19 vaccines. We aimed to describe our experience in dealing with people residing in Qatar from the perspective of healthcare practitioners from the Qatar University Health Center during the period when mRNA COVID-19 vaccines was introduced in a time frame of 6 months (April to October, 2021).

We identified several factors associated with the reluctance to receive mRNA COVID-19 vaccines once vaccination services were available, affordable, and accessible to everyone in Qatar (Table 1). Most individuals were hesitant and refused to take mRNA COVID-19 vaccines owing to the unjustified myths and fear about potential side effects of vaccines in general and unknown long-term effects of vaccination, especially among women who were uneducated. We believe we have been able to put forth a fair, unbiased, and balanced argument between an individual's right to take or refuse the vaccine and the overall benefits to the public and community health in terms of the overall community immunity when the vast majority of the population will be vaccinated. Our experience could assist in developing culturally sensitive and tailored community outreach programs to increase community awareness as it is the cornerstone on which public health can fight the irrational myths, fear, misconceptions, vaccine hesitancy, and improve vaccination coverages. Moreover, our shared experiences might be able to better prepare future launching of pandemic vaccination campaigns in order to minimize vaccine hesitancy.

## Figures and Tables

**Table 1:** Factors affecting mRNA COVID-19 vaccine hesitancy at the Qatar University Health Center

**#**	**Factor**	**Explanation**

1.	**Speedy preparation and FDA approval for mRNA COVID-19 vaccines**	• Many patients reported that their hesitancy to receive the mRNA COVID-19 vaccine originated from the fact that the vaccine was developed rapidly with no evidence of potential consequences.• Many patients reported legitimate concerns and uncertainties about the speedy production process, safety, and efficacy of these newly developed vaccines.

2.	**Individual and social factors**	• Being a woman was associated with greater hesitancy to receive the vaccine.• Most female patients shared and reported their fear and concerns around the future consequences of mRNA COVID-19 vaccines as they perceived it to affect their fertility and increase the chances for future diseases among their children.

3.	**Different types of COVID-19 vaccines used in the country**	• Patients frequently reported concern about the safety and efficacy of the available vaccine options, including mRNA COVID-19 vaccines.• We noticed a sense of uncertainty that increased anxiety in terms of “which vaccine was better for me?”• Patients frequently questioned the benefits and value of taking a specific vaccine type based on the country of origin.• Patients questioned the effect on their friends or relatives within the country or across the world.• Patients commented on health equity and equality regarding receiving a specific type of vaccine stemming from their misconception and the proactive listening to the myths around the effect of each type of available vaccine.• However, many patients also reported feeling “grateful” for the availability of vaccine services and free vaccine even if they were not Qatari.

4.	**Vaccine safety**	• Vaccine safety was commonly reported as one of the main reasons for vaccine hesitancy with all vaccines, including mRNA COVID-19 vaccines and particularly, the potential future consequences of these vaccines.

5.	**The impact of social rumors, myth, media, and the readiness of healthcare providers to address vaccine hesitancy**	• Many participants strongly believed that there was no pandemic, and the spread of the pandemic was part of a hidden business or political agenda or a marketing strategy to sell useless and meaningless vaccines.• These patients frequently believed some of the conspiracy theories around the vaccine. For instance, some patients reported that having a vaccine can be one way to insert plates or microchips inside their bodies so people can track them biologically.• Therefore, providing front-line and healthcare professionals with proper training, including communication skills, different relational approaches, and access to evidence-based and tailored information on the different COVID-19 vaccines, can positively affect their discussion with patients.


